# Uniform and bright light emission from a 3D organic light-emitting device fabricated on a bi-convex lens by a vortex-flow-assisted solution-coating method

**DOI:** 10.1038/s41598-019-54820-9

**Published:** 2019-12-03

**Authors:** Byoungchoo Park, Seo Yeong Na, In-Gon Bae

**Affiliations:** Department of Electrical and Biological Physics, Kwangwoon University, Wolgye-Dong, Nowon-gu Seoul, 01897 Republic of Korea

**Keywords:** Organic LEDs, Design, synthesis and processing, Photonic devices, Surfaces, interfaces and thin films

## Abstract

We herein present the results of a study on the novel fabrication process of uniform and homogeneous semiconducting polymer layers, in this case hole-injecting and fluorescent light-emitting layers that were produced by a simple solution-coating process for 3D conformal organic light-emitting diodes (3D OLEDs) on curvilinear surfaces. The solution-coating process used was a newly developed method of vortex-flow-assisted solution-coating with the support of spinning of the coating solution. It is shown that the vortex-flow-assisted spin-coating process can produce high-quality thin films at nanoscale thicknesses by controlling the liquid surface of the coating solutions, which can easily be adjusted by changing the spinning speed, even on complex curvilinear surfaces, i.e., a quasi-omnidirectional coating. This excellent film-forming ability without any serious film defects is mainly due to the reduction of line tension among the solution, air, and the substrate at the contact line due to vortex flows of the coating solution on the substrate during the vortex-spin-coating process. As a proof of concept, we present vortex-spin-coated 3D OLEDs fabricated on bi-convex lens substrates which exhibit excellent device performance with high brightness and current efficiency levels comparable to those of a conventional spin-coated 2D planar OLED on a flat substrate. It is also shown that the EL emission from the 3D OLED on the bi-convex lens substrate exhibits a diffusive Lambertian radiation pattern. The results here demonstrate that the vortex-flow-assisted spin-coating process is a promising approach for producing efficient and reliable next-generation OLEDs for 3D conformal opto-electronics.

## Introduction

Organic/polymeric semiconducting materials have attracted much interest from both academic and industrial researchers as these materials have several significant advantages, such as their high degree of flexibility, lightness, and simple processing capabilities, especially for flexible/plastic electronics^1–10^. Nearly all the organic/polymeric semiconducting materials utilised for electronic applications, such as organic light-emitting diodes (OLEDs)^1–3^, organic photovoltaics (OPV)^4–6^, organic thin film transistors (OTFTs)^7–10^, organic lasers^11,12^, and organic volatile memory devices^13,14^, are prepared as thin film structures on the nanometer scale. Hence, the fabrication of uniform thin films on the nanoscale is an extremely important process that must be controlled precisely.

In terms of the fabrication process, organic/polymeric thin films have typically been prepared by low-temperature deposition techniques from coating solutions. Examples include spin coating^3–5^, screen printing^15,16^, bar-coating^17^, blade coating^18,19^, slot-die coating^20,21^, horizontal-dip coating^22,23^, inkjet printing^24^, or brush coatings^25^, together with the vacuum thermal evaporation method^1,2^. The most of these methods can produce uniformly thin layers of organic/polymeric materials mainly on a flat and planar substrate, such as a silicon wafer or glass plate, and several advantages of these coating techniques have been documented. Nevertheless, these coating techniques are unsuitable for the fabrication of uniform layers on the curvilinear substrates of 3D objects, especially for 3D conformal electronics, which will make up the key components of the next generation of microelectronics by integrating electronic systems with a complex topography^26,27^.

Recently, to overcome the drawbacks of 2D planar electronics, several fundamental and conceptual advances in fabrication processes together with new materials and designs have been developed, such as micro-contact printing^28^, nanotransfer printing^29^, direct imprinting^30,31^, inkjet printing^32^, 3D (extrusion-based) printing^27,33^, pad-printing^34^, and electrospray deposition^35,36^. These techniques can be used to produce interesting 3D electronic systems, exhibiting fundamentally new characteristics for innovative applications in various fields, such as wearable electronics, conformal displays, energy harvesters, bio-medical electronics, healthcare, advanced optical, and/or multimodal sensing systems^26,27^. Among them, electrospray deposition, utilizing an electrical field that disrupts the liquid drops and causes them to atomize, may allow the deposition of a thin film on a curvilinear surface^35,36^. As a recent example, by using the electrospray deposition technique, thin functional layers were fabricated on a concave glass substrate to obtain 3D OLEDs^36^. However, the electrospray-deposited layers in 3D OLEDs are not sufficiently uniform and homogeneous to produce uniform electroluminescent (EL) light emission as well as high device performance^36^. Thus, the uniform deposition of a functional thin film on a complex curvilinear surface in the nanoscale thickness range remains a challenge and the development of an alternative solution process by which to fabricate a uniform thin layer on a curvilinear substrate is an interesting goal which could expand the use of such a substrate to new opto-electronics, forming the basis for the next generation of 3D conformal opto-electronics.

We herein describe the use of a novel solution process assisted by the spinning of a vortex flow of a coating solution to fabricate uniform thin layers on 3D curvilinear surfaces as well as conventional 2D planar surfaces. (Hereafter, we refer to the proposed process as the vortex-spin-coating process.) It is shown that the vortex-spin-coating process can produce highly uniform and fully thickness-controllable thin layers on 3D substrates by spreading a contact line of a liquid withdrawn by a vortex flow of a coating solution on a curvilinear surface. The surface morphology investigation conducted here shows that the topography of the vortex-spin-coated layer on a bi-convex lens substrate is fairly uniform with no serious defects, such as poor coverage, pin holes, cracks, wrinkles, or crinkles. It was also found that the surface thickness of the vortex-spin-coated films on the lens was identical at different positions. This excellent film-forming ability is mainly due to the reduced line tension levels among the solution, air, and substrate at the contact line during the coating process, in contrast to the typical coating methods mentioned above. Moreover, the film thickness of the vortex-spin-coated layer can be explained in terms of a typical spin-coating description^37–39^. As a proof of concept, we present vortex-spin-coated 3D OLEDs fabricated on bi-convex lens substrates which exhibit excellent device performance with a solution-processable hole-injecting polymer (poly (styrene sulfonic acid) doped poly (3, 4-ethylenedioxythiophene), PEDOT:PSS) as a hole-injecting layer (HIL) and a yellow-emitting copolymer (Super yellow, known as SY) as a light-emitting layer (EML). Considering the significant difficulty encountered when fabricating uniform thin layers on complex 3D substrates, these results reveal the important contribution of vortex flows during the solution-coating process. The advances described here can thus pave the way towards 3D conformal electronics; furthermore, the interesting performance capabilities of these devices as a 3D OLED and/or 3D lighting creation also add to their appeal.

## Results and Discussion

### The vortex-flow-assisted spin-coating process

**Figure 1:**
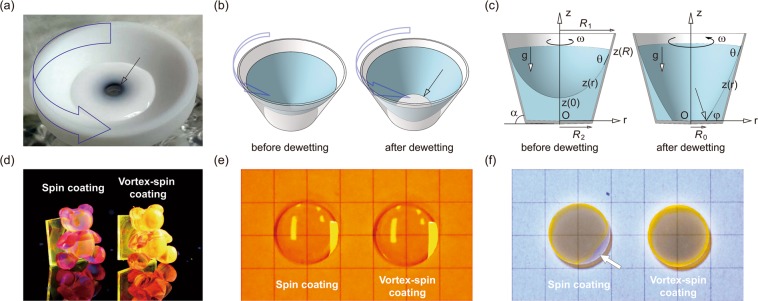
Vortex-spin-coating of functional material solutions and their layers coated on curvilinear surfaces. Photograph (**a**) and schematic illustration (**b**) of the vortex-assisted spin-coating method with a rotating truncated conical chuck containing a substrate (arrow) mounted at the centre of the bottom surface and a coating solution exhibiting a vortex flow of the coating solution. In the photograph, a white aqueous solution was used as a coating solution to show the vortex flow clearly. (**c**) Left: a depiction of the axisymmetric surface shape of a solution in a uniform rotating truncated conical container of inner radius *R*_1_ (upper side) and *R*_2_ (bottom side) with an angular speed of ω. The coordinate origin is at the centre of the bottom surface. The height of the liquid from the coordinate origin is *z*(0). Right: the liquid surface shape after the onset of dewetting, which has spread to the radius *R*_0_ (arrow). The wetting contact angles at the bottom and the lateral wall of the container are denoted by *φ* and *θ*, respectively. (**d**) UV PL photograph of two plastic toys coated with fluorescent SY layers by the conventional spin-coating (left) and the vortex-spin-coating (right) methods under UV light illumination (365 nm). Photograph (**e**) and UV PL photograph (**f**) of two bi-convex lenses (diameter = 9.5 mm, focal length = 20 mm) coated with a fluorescent SY layer by the conventional spin-coating (left) and the vortex-spin-coating (right) methods. The arrow shows an uncoated edge region of the lens substrate.

A photograph and schematic illustrations of the vortex-flow-assisted spin-coating process under investigation are shown in Figs. 1a,b, respectively. These figures show a rotating truncated conical chuck containing a substrate attached at the centre of its bottom surface with a vortex flow of a coating solution over the substrate. In a rotating container (e.g., a cylinder), it is known that the surface shape of a liquid solution can be divided into two categories, i.e., before or after dewetting of its bottom surface, according to whether the liquid surface exhibits a simple hyperbolical dimple of a vortex or approaches the bottom surface of the container^40^. After dewetting, i.e., when the liquid withdraws from the bottom surface of the container (or the top surface of the substrate), a thin liquid film can be deposited onto the bottom surface (or the top surface of the substrate) behind the withdrawing liquid with a radius *R*_0_ (Fig. 1c and Supplementary Fig. S3). The vanishing line tension (τ* = 0, see Eq. (S3.12)) at *R*_0_ can indicate that the liquid layer can evenly coat the substrate surface, resulting in little variation of the thickness of the deposited layer, even on a 3D curvilinear substrate surface.

Thus, we exploited the dewetting effect of the vortex-flow-assisted spin-coating process, which has the following sequence. (1) The substrate is attached to the bottom surface of the truncated conical chuck at its centre. A coating solution of a functional material is then used to fill the chuck to cover the substrate completely so that a flat uniform liquid surface of the solution can be formed over the substrate before its rotation. (2) The chuck and the substrate are then rotated at a specific angular velocity ω whilst generating a vortex flow and controlling the surface shape of the coating solution (spin-up stage). After the onset of dewetting, a thin layer of the solution is then deposited evenly on the substrate behind the withdrawing solution, spreading to radius *R*_0_, at which line tension vanishes (τ* = 0, Eq. (S3.12)) along the triple contact line (*R*_0_) on the bottom surface with a zero contact angle (*φ* = 0, Eq. (S3.13)) and zero slope (*z*′(*R*_0_) = 0, Eq. (S3.14)) of the liquid surface profile (Fig. 1c and Supplementary Fig. S3). While the substrate is being rotated, the radius *R*_0_ of the withdrawing solution increases and finally becomes larger than the size of the substrate (deposition stage). A thin liquid layer is thus formed over the entire surface of the substrate by the complete dewetting of the solution. Subsequently, angular velocity ω increases further, with the remaining solution then spilling over the chuck. Thus, after the rotation of the substrate, the substrate is not re-wetted and disturbed by the remaining solution (spin-off stage). (3) Having been coated onto the substrate, the liquid layer is dried and a heater can be used to promote the evaporation of the residual solvent in the liquid layer on the substrate (evaporation stage). Following this process, it is possible to obtain a substrate coated with a thin solid film with a uniform thickness. The rotation of the substrate during the coating process is somewhat similar to how this is done when using the typical spin-coating method, but it is noteworthy that the vortex-spin-coating method differs from the conventional spin-coating method, especially with regard to dewetting by the vortex flow of the coating solution, spreading the coating solution uniformly onto the rotating substrate.

First, we examined the vortex-spin-coating of a fluorescent polymer solution of SY as a test material on small toys. They were fixed on glass substrates for attachment to the bottom surface of the conical chuck. Figure 1d shows a photoluminescence (PL) image obtained from typical spin-coated and our vortex-spin-coated SY layers on the toys. This figure shows that the PL intensity of the SY layer spin-coated onto the irregular surface of the toy significantly varies at nearly every part of the toy surface (see the toy on the left of the figure). On the other hand, interestingly, it is also apparent that the vortex-spin-coated layer exhibits highly uniform and bright PL intensity over the entire surface of the toy (see the toy on the right of the figure). Variation in the PL intensity of the vortex-spin-coated SY layer on the toy was observed only at the very rear edge of the toy. This indicates that the vortex-spin-coating process provides a uniform coating of functional material solutions even on a complex topography, such as nonplanar, uneven, and polyhedron surfaces, thus providing quasi-omnidirectional coatings of functional materials.

Next, in order to estimate the quality of the film coated onto a 3D substrate, we used a bi-convex lens (diameter = 9.5 mm, radius of curvature = 20 mm) as a test 3D curvilinear substrate. We observed the macroscopic surface morphologies of the solution-coated fluorescent SY layers on the bi-convex lenses, as shown in Fig. 1e,f. In these figures, we compared typical macroscopic photographic and UV PL images of the two lenses coated with SY layers using the typical spin-coating and vortex-spin-coating methods. As is clear from the photograph in Fig. 1e, the two lenses coated with SY layers appear to be nearly uniform and identical to each other, not only at the centre region but also at the edge region. However, Fig. 1f clearly indicates that the UV PL image of the spin-coated SY layer varies near the edge of the lens substrate with a large defect in the layer (see the arrow in the figure). This defect of poor coverage is due mainly to the imperfect substrate wetting and/or difficulty in the uniform dispensing of the liquid onto the curvilinear surface when using the conventional spin-coating method. In order to reduce these types of defects of the spin-coated layer, a large amount of coating solution may be deposited onto the lens surface; however, overflow of the solution on the lens surface also frequently induces surface defects of the coated layer. Thus, the area of the uniform SY layer is smaller than the size of the lens substrate and is usually limited at the centre region of the lens, as shown in the UV photograph. In contrast to the inhomogeneous spin-coated SY layer, the UV PL image shows that the SY layer vortex-spin-coated onto the lens is fairly uniform over nearly the entire surface of the lens substrate, with the only exception being the edge beads. This uniformity is mainly achieved by the negligible line tension (τ* ~ 0) applied during the vortex-spin-coating of the SY solution onto the substrate, as mentioned above.

**Figure 2:**
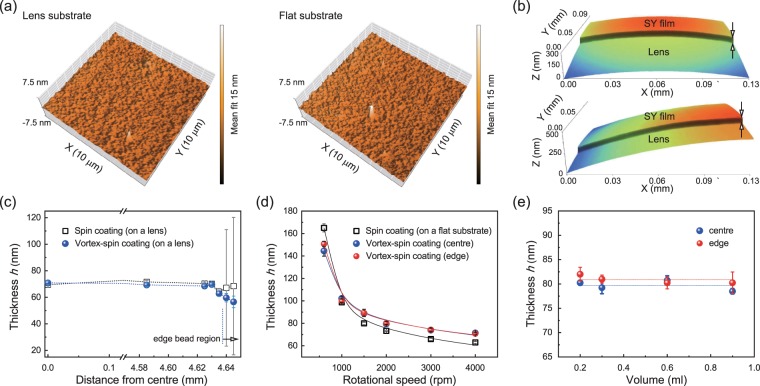
Surface morphologies of the vortex-spin-coated layers on bi-convex lens substrates and their film thickness dependences. (**a**) AFM topographic images of the vortex-spin-coated SY layer on a bi-convex lens (left) and typical spin-coated SY layer on a flat substrate (right). (**b**) Non-contact 3D optical microscopic images of cross-sections of the SY layer fabricated by the vortex-spin-coating method on the lens at its centre (upper) and on the edge (lower). (**c**) Dependence of the layer thickness as a function of the radial distance from the centre for the vortex-spin-coated SY layers and for the typical spin-coated layers on bi-convex lens substrates at the given spinning speed (2000 rpm). (**d**) Dependence of the layer thickness as a function of the rotational speed ω for vortex-spin-coated SY layers on bi-convex lens substrates and for typical spin-coated layers on flat glass substrates. Solid curves show the theoretical fits. (**e**) Dependence of the SY layer thickness as a function of the volume of the coating solution in the conical chuck for vortex-spin-coating at the given spinning speed (2000 rpm). Dotted lines show the averaged values of the measured film thicknesses.

Next, we investigated the microscopic surface morphology characteristics of the fabricated SY layers using non-contact AFM measurements and the 3D surface profiler (Fig. 2). The panel on the left-hand side in Fig. 2a presents a topographic AFM image of the vortex-spin-coated SY layer at the centre region of the bi-convex lens substrate, showing a fairly smooth surface with a RMS roughness of ~0.33 nm. For comparison, we also created a SY layer formed on a 2D flat substrate using the typical spin-coating method, as shown on the panel on the right-hand side in Fig. 2a. The spin-coated SY layer presents an AFM morphology with an RMS roughness of ~0.34 nm, similar to that of the vortex-spin-coated SY layer. For another comparison, we obtained microscopic scanning electron microscopy (SEM) images of vortex-spin-coated SY layers on lens surfaces (Supplementary Fig. S4). The surface morphology as investigated using SEM confirmed that the topography of the vortex-spin-coated SY layer even on the bi-convex lens substrate was fairly uniform without any serious defects and/or pin-holes, clearly similar to the topography of a typical spin-coated SY layer on a flat substrate.

We also compared representative microscopic cross-sectional images of the vortex-spin-coated SY layer at the centre and edge regions of the lens surfaces using the 3D surface profiler (Fig. 2b). The surface morphology investigation using the 3D profiler showed clearly that the topography of the SY layer was fairly uniform with a RMS roughness of less than ca. 1.0 nm, which was clearly similar to that (ca. 1.0 nm) of the typical spin-coated SY layer on a flat substrate (not shown). These results confirm that the vortex-spin-coated SY layer is highly uniform and homogeneous without any noticeable aggregation and/or film defects, such as poor coverages, pin holes, or cracks in the coated layer. It was also noted that the nearly identical surface roughness and uniformity of the vortex-spin-coated SY layers indicate few or no differences in their film properties relative to those of the conventional spin-coated layers on a 2D flat substrate. Moreover, the film thickness *h* of the vortex-spin-coated SY layer was nearly identical at different positions on the lens surface, in contrast to the spin-coated layer, especially for the edge region, as shown in Fig. 2c. Here, it is noted that in the figure, the edge regions show large deviations of the thickness values due to the beginning of the edge bead regions and/or defects, especially for the spin-coated layer. Accordingly, further edge bead regions were omitted in the figure for a clear comparison. Thus, compared to the typical spin-coating method, it is possible to achieve high uniformity of the film thickness for the coated layer reliably by using the vortex-spin-coating method, even on a 3D curvilinear substrate.

Next, we investigated the dependence of the film thickness *h* of the vortex-spin-coated SY layer on the rotational speed ω and on the volume *V*_0_ of the coating solution in the conical chuck. The corresponding results obtained from the films coated onto the lens substrates are shown in Figs. 2d,e. As shown in Fig. 2d, for a given volume of the solution *V*_0_ of 0.2 ml, the film thickness *h* directly corresponds to the rotational speed; i.e., the thickness *h* of the vortex-spin-coated SY layer decreases continuously as the angular speed (ω) increases in both the centre and edge regions of the lens substrate. This result is similar to the theoretical description of the conventional spin-coating model, which can be explained using the empirical equation (*h* ∝ a(1 + b(ω^2^ + c))^−d^)^37–39^. The theoretical curves from the equation are shown in the figure as solid curves. The observed data are well fitted to the values predicted by the equation, indicating that the thickness of the vortex-spin-coated layer follows a trend nearly identical to those of the typical spin-coated layers. For comparative purposes, the experimental results with a *V*_0_ value of 0.1 ml and fitted theoretical curves for typical spin-coated SY layers on flat glass substrates are also shown in the figure. From this comparison, it is evident that the thickness of the vortex-spin-coated SY layer can be suitably controlled by adjusting the spinning speed. Furthermore, when *V*_0_ was increased from 0.2 ml to 0.9 ml, the thickness of the vortex-spin-coated SY layer is nearly independent of *V*_0_ for both the centre and edge regions of the lens substrate, as shown in Fig. 2e. These results indicate that the vortex-spin-coating process can successfully produce uniform and thin functional layers on 3D curvilinear surfaces at least as well as conventional spin coating can on 2D planar surfaces.

**Figure 3:**
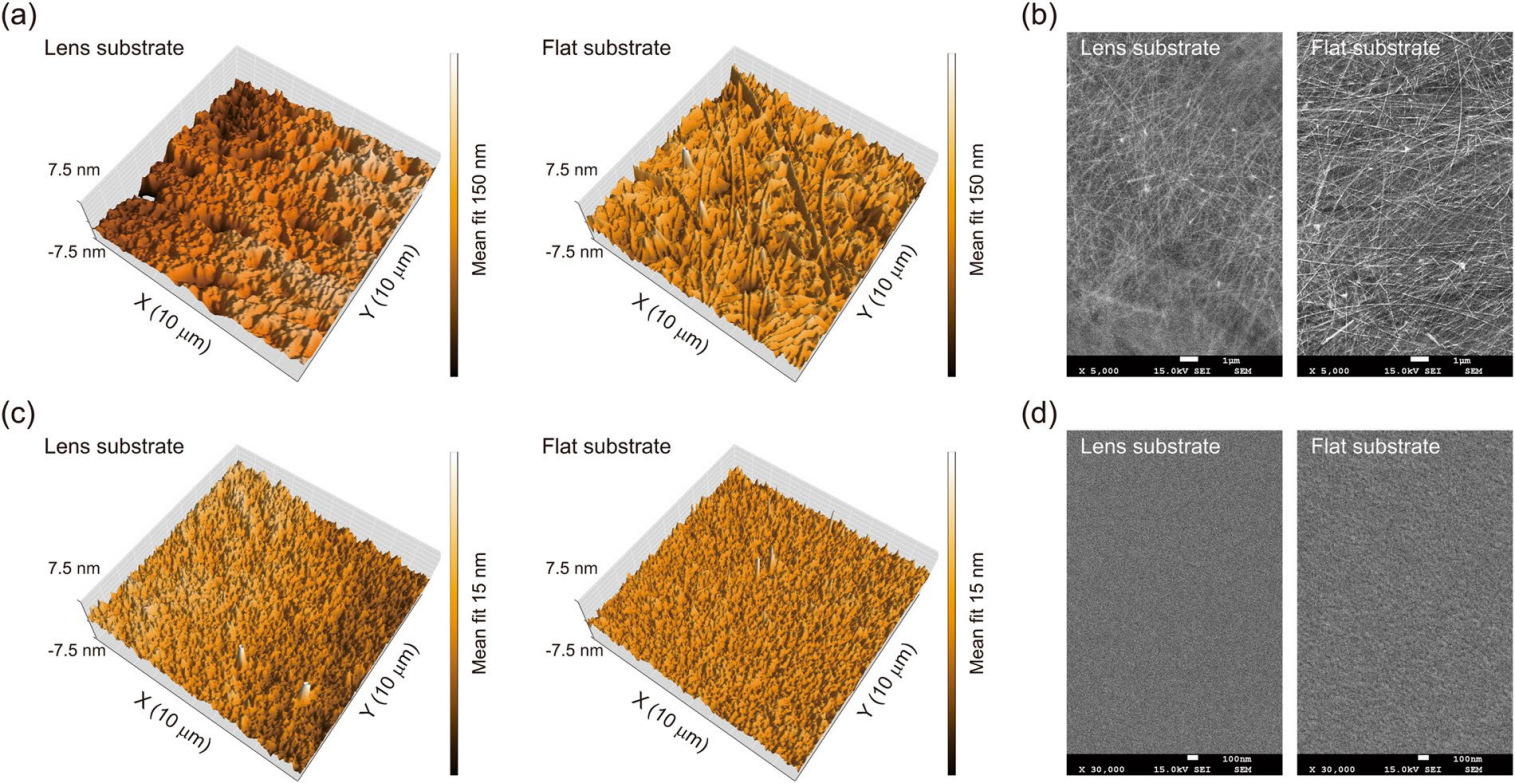
Surface morphologies of vortex-spin-coated functional layers on bi-convex lens substrates and corresponding scanning electron microscopy (SEM) images. (**a**) AFM topographic images of the vortex-spin-coated Ag-NW layer on a bi-convex lens (left) and a typical spin-coated Ag-NW layer on a flat substrate (right). (**b**) SEM images of Ag-NW layers fabricated by the vortex-spin-coating method on the lens at its centre (left) and by the conventional spin-coating method on a flat substrate (right). (**c**) AFM topographic images of the vortex-spin-coated PEDOT:PSS layer on a bi-convex lens (left) and a typical spin-coated PEDOT:PSS layer on a flat substrate (right). (**d**) SEM images of the PEDOT:PSS layers fabricated by the vortex-spin-coating method on the lens at its centre (left) and by the conventional spin-coating method on a flat substrate (right).

Next, we also fabricated other functional layers, in this case a transparent silver nanowire (Ag-NW) electrode and a PEDOT:PSS HIL, on convex lenses with the vortex-spin-coating process and investigated the characteristics of the microscopic surface morphology of the fabricated layers using non-contact AFM and SEM measurements (Fig. 3). The panel on the left-hand side in Fig. 3a (Fig. 3c) presents a topographic AFM image of the vortex-spin-coated Ag-NW (PEDOT:PSS) layer at the centre region of the bi-convex lens substrate, showing a fairly smooth surface with a RMS roughness of ~10.0 nm (0.85 nm). For comparison, we also fabricated an Ag-NW (PEDOT:PSS) layer on a 2D flat substrate using the typical spin-coating method, as shown on the panel on the right-hand side in Fig. 3a (Fig. 3c). The spin-coated Ag-NW (PEDOT:PSS) layer presents an AFM morphology with an RMS roughness of ~11.6 nm (0.72 nm), similar to that of the vortex-spin-coated layers. We also compared typical microscopic SEM images of the vortex-spin-coated layers at the centre regions of the lens surfaces (the left-hand sides in Fig. 3b,d). This investigation of the surface morphology with SEM showed clearly that the topographies of the vortex-spin-coated functional layers on the bi-convex lens substrates were fairly uniform, clearly similar to the topographies of the typical spin-coated layers on flat substrates (the right-hand sides in Fig. 3b,d). These results confirm that the vortex-spin-coated functional layers such as the Ag-NW layer and the PEDOT:PSS layer, are also highly uniform and homogeneous without any noticeable film defects, such as poor edge coverages, pin-holes, or cracks in the coated layer. Moreover, the nearly identical surface roughness and uniformity of the vortex-spin-coated functional layers present evidence of few or no differences in their film properties relative to those of the conventional spin-coated layers on a 2D flat substrate. The film thicknesses of the vortex-spin-coated functional layers were also nearly identical at different positions on the lens surface. More details about the Ag-NW and PEDOT:PSS functional layers will be reported elsewhere. Thus, it is possible to achieve highly uniform and reliable functional layers of Ag-NW and PEDOT:PSS by using the vortex-spin-coating method, even on a 3D curvilinear substrate.

### 3D OLEDs on bi-convex lens substrates

**Figure 4:**
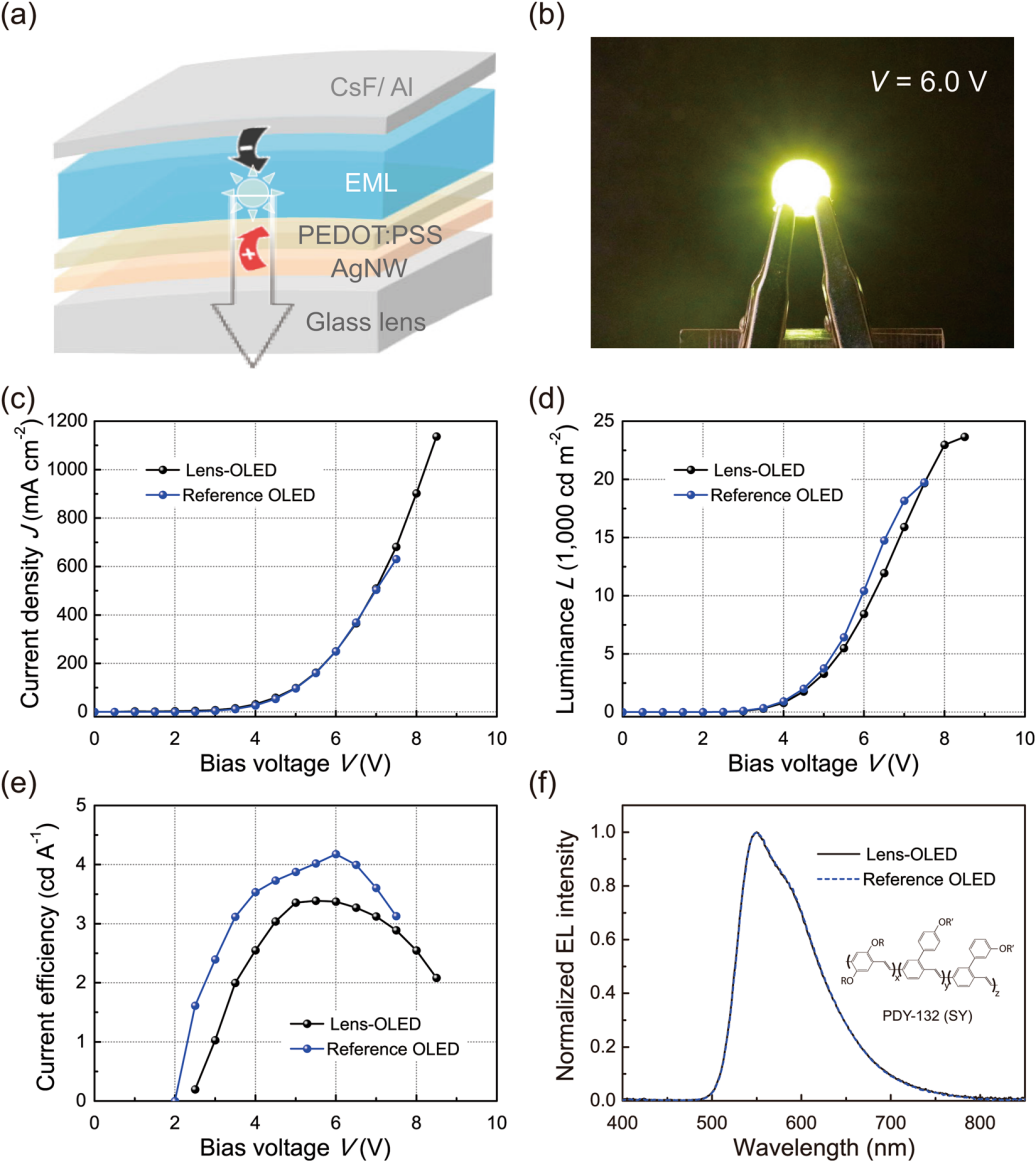
3D OLEDs fabricated on bi-convex lens substrates by the vortex-spin-coating method. (**a**) Schematic illustration of the device architecture of a 3D OLED on a bi-convex lens substrate (Lens-OLED) with an Ag-NW anode, a PEDOT:PSS HIL, a SY EML, a CsF EIL, and an Al cathode. (**b**) Photograph of a Lens-OLED sample with the vortex-spin-coated PEDOT:PSS HIL and SY EML operating at 6.0 V, demonstrating bright EL emission from the Lens-OLED (active area: 3 × 3 mm^2^). Current density-voltage (*J*-*V*) (**c**), luminance-voltage (*L*-*V*) (**d**), and luminance efficiency-voltage (*LE*-*V*) (**e**) characteristics of the Lens-OLED and a flat reference OLED. (**f**) Normalised EL spectra measured at the surface-normal direction for the Lens-OLED (black) sample and for the reference OLED (blue) operating at 6.0 V. The inset in (**f**) shows the molecular structures of SY used in the EML.

Given the impressive film quality levels of the functional layers fabricated by the vortex-spin-coating technique, we fabricated OLEDs using a PEDOT:PSS HIL and a SY EML on a bi-convex lens (Lens-OLED) to investigate whether the vortex-spin-coated layers were electronically functional. For the Lens-OLEDs, yellow-emitting devices with a structure of [Ag-NW anode/PEDOT:PSS HIL/SY EML/CsF electron-injecting layer (EIL)/Al cathode] were fabricated, as shown in Fig. 4a. We emphasise that the Ag-NW anode, PEDOT:PSS HIL and SY EML were vortex-spin-coated subsequently for this device. For comparison, a reference device with a typical spin-coated PEDOT:PSS HIL and a SY EML on a flat glass substrate was also fabricated. Figure 4b shows a photograph of an operating Lens-OLED sample fabricated at the centre of a bi-convex lens surface. The Lens-OLED generated bright EL light at 6.0 V, exhibiting excellent EL light-emission from the Lens-OLED with the vortex-spin-coated functional layers of the SY EML and PEDOT:PSS HIL.

In order to evaluate the opto-electronic characteristics of the vortex-spin-coated PEDOT:PSS HIL and SY EML with regard to the device performance level, we investigated the current density-voltage-luminance (*J*-*L*-*V*) characteristics of the fabricated Lens-OLEDs, as shown in Fig. 4c,d. For the Lens-OLED, the slopes of the *J*-*V* curves between 0 and 9.0 V indicated excellent diodic behaviours of the vortex-spin-coated layers (Fig. 4c). It is also clear from the *J*-*L*-*V* curves (Fig. 4c,d) that the charge injections are well below 2.5 V, with sharp increases in the *J*-*L*-*V* curves above this range. For example, the operating voltage of the Lens-OLED with the vortex-spin-coated layers on a bi-convex lens substrate is approximately 3.2 V for a brightness of 100 cd/m^2^; it is 4.2 V for 1,000 cd/m^2^ and 6.2 V for 10,000 cd/m^2^. The maximum luminescence reached ca. 24,000 cd/m^2^ (at 8.5 V). This result shows that the EL brightness levels are significantly high in the Lens-OLED with the vortex-spin-coated functional layers. For comparison, the operating voltage of the reference OLED with the spin-coated layers on a flat substrate is close to 3.0 V for a brightness of 100 cd/m^2^; it is 4.2 V for 1,000 cd/m^2^ and 6.0 V for 10,000 cd/m^2^. The luminescence reached ca. 20,000 cd/m^2^ (at 7.5 V). Interestingly, it was also noted that the efficiency of the Lens-OLED is comparable to that of the flat reference device, even for the curved vortex-spin-coated layers (Fig. 4e); the peak luminance efficiency (*LE*_max_) of Lens-OLED is 3.4 cd/A, which is comparable to that (4.2 cd/A) of the reference device with the flat spin-coated layers. Even at a high luminance level of 20,000 cd/m^2^, the *LE*s of the sample devices with the vortex-spin-coated layers reach 2.9 cd/A, which is also comparable to that (3.1 cd/A) of the reference OLED. For another comparison, a comparative OLED based on an ITO anode with spin-coated layers on a flat substrate was found to achieve a peak brightness of ~31,000 cd/m^2^ and peak current efficiency of ~5.8 cd/A. In another comparison, we also investigated the device characteristics of OLEDs fabricated by the conventional spin-coating method on lens substrates (Supplementary Fig. S5). These OLEDs with typical spin-coated SY layers on the lenses exhibited large variations in their *J-V* and *L-V* characteristics mainly due to the low reproducibility of the formation of a uniform film on the lens substrates, in contrast to the fairly reproducible device characteristics of the OLEDs with the vortex-spin-coated SY layers on the lenses. We also investigated the EL spectra of the fabricated OLEDs in the surface-normal direction. Figure 4f shows the normalised EL spectra of the Lens-OLED and flat reference OLED (at 1,000 cd/m^2^). As shown in the figure, characteristic dominant EL spectral peak is observed at ~550 nm with a full-width at half-maximum (FWHM) of ~95 nm for the Lens-OLED, nearly identical to that of the reference OLED and similar to that of the comparative OLED. The device performances of the Lens-OLED studied here are summarised in Table 1.

**Table 1:** EL characteristics of the fabricated OLEDs.

Characteristics	Lens-OLED	Reference OLED	Comparative OLED
Anode/substrate	AgNW/3D lens	AgNW/2D glass	ITO/2D glass
*V*_ON_ (V)	2.3	2.5	2.5
*L*_max_ (cd/m^2^) (V at *L*_max_)	24,000 (8.5)	20,000 (7.5)	31,000 (10.0)
*LE*_max_ (cd/A)	3.4	4.2	5.8
λ_max_ (nm)	551	551	547
FWHM (nm)	95	95	80

*V*_ON_: turn-on voltage, *L*_max_: maximum luminance, *LE*_max_: maximum current efficiency, λ_max_: emission peak wavelength.

Thus, the comparably high EL brightness and efficiency of the Lens-OLED to those of the reference OLED indicate that excitons are efficiently generated by the introduction of the vortex-spin-coated layers, even on the curvilinear substrate, which may cause a number of radiative exciton recombinations via the electron-hole balance in the EMLs. A possible mechanism responsible for the efficient formation of excitons is the combination of good coverage without film defects and balanced electron or hole current flows in the vortex-spin-coated layers. The results above clearly indicate that the device performance levels were well implemented when the vortex-spin-coated layers were introduced on the curvilinear surface of the lens. Further improvements in the device performance of the Lens-OLED are possible by selecting appropriate materials, controlling the layer thickness, undertaking processing optimization steps, and by introducing an inert coating environment.

**Figure 5:**
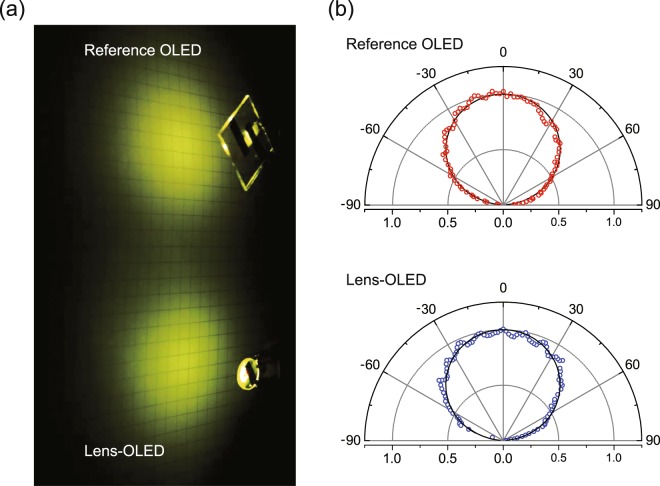
EL emission patterns of the 3D OLED. (**a**) Photographs of the radiation patterns of the reference OLED (upper) and the Lens-OLED samples (lower) at a distance *d* = 4.0 cm between the device and the screen used. (**b**) Polar plot of the viewing angle dependence of the EL intensity from the reference OLED (upper) and the Lens-OLED (lower) when *d* = 4.0 cm. The solid curves represent the Lambertian emission pattern.

Next, we investigated the EL emission patterns of the fabricated OLEDs, as shown in Fig. 5. Figure 5a shows the radiation patterns of the devices at a distance *d* of 4.0 cm between the device and the screen used. As shown in the figure, the reference device shows a well-known diffusive emission pattern, following the Lambertian law^41^, as clearly shown in the upper panel in Fig. 5b. Interestingly, the Lens-OLED also exhibits an EL emission pattern similar to that of the reference device (lower panel in Fig. 5b). These outcomes clearly indicate that the Lens-OLED is a feasible 3D OLED for various 3D lighting applications. Additional detailed information pertaining to the emission patterns is beyond the scope of this report and will be reported elsewhere.

The foregoing results clearly show that the light-emitting performance of the 3D Lens-OLED fabricated by the vortex-spin-coating method is comparable to that of the conventional OLEDs on 2D flat substrates, demonstrating that the utilization of vortex-spin-coated functional layers into 3D objects readily produces bright and efficient 3D OLEDs. To the best of our knowledge, this is the first demonstration of high device performance of a 3D OLED, exhibiting high brightness and a high current efficiency. Therefore, considering the difficulty encountered when fabricating functional layers on curvilinear substrates, the simple vortex-spin-coating process for OLEDs as introduced here clearly presages the development of inexpensive, fast, large-area, and high-performance 3D light-emitting devices and new 3D lighting creations. Furthermore, we envision that the vortex-spin-coating of 3D-OLEDs on such lenses may potentially be used for wearable smart contact lenses for on-eye sensors or displays.

Finally, it is important to comment on the Ag-NW anode on the bi-convex lens surface used in this study, which was fabricated by the transfer method^42^. To transfer Ag-NWs onto the lens surface, an Ag-NW suspension was also coated onto a concave substrate using our vortex-spin-coating method, after which the coated Ag-NW layer on the concave surface was transported to the lens surface. Thus, the vortex-spin-coating method is also very useful for the fabrication of transparent Ag-NW electrodes on curvilinear surfaces. More details about the process used to transfer the Ag-NWs will be reported elsewhere. It should also be noted that the vortex-flow-assisted solution coating is useful for fabricating thin, uniform, and homogeneous films for various types of materials, such as functional small molecules and nano-sized functional materials. However, examinations of such outcomes are beyond the scope of the current study, Therefore, further details about the fabrication of such layers and related discussions will be reported elsewhere.

## Conclusions

In summary, we introduced a simple vortex-flow-assisted solution-coating method as a promising process for next-generation 3D OLEDs. It is shown that our vortex-spin-coating process allows for critical control of the uniform and homogeneous formation of a functional layer, even on complex curvilinear substrates at speeds up to a few thousand rpm. We verified that this process can produce high-quality thin films on curvilinear surfaces by utilizing the dewetting behaviour of the vortex surface of the coating solution, which can be controlled by adjusting the important parameter of the spinning speed. We contend that the reduction of the line tension of the coating solution due to the vortex flows of the solution on the substrate is crucial for the formation of thin films without any serious film defects. On the basis of these outstanding film quality levels of the vortex-coated layers together with the simple processability presented here, we successfully produced a proof-of-concept device of a 3D OLED by combining charge-carrier injecting and light-emitting functional layers with the versatility of the vortex-spin-coating technique. It was demonstrated that the device performance capabilities of the 3D OLEDs created on bi-convex lens substrates are comparable to those of conventional spin-coated OLEDs on 2D flat substrates. It can therefore be confirmed that this novel coating process is promising for achieving highly uniform and homogeneous functional layers on complex curvilinear substrates of 3D objects for 3D conformal electronics and that it will provide a solid foundation for realizing the fabrication of efficient, reliable, and flexible 3D opto-electronic devices, including advanced 3D conformal displays. Further, this vortex-spin-coating technique is easily expandable to the fabrication of other classes of 3D electronics, including wearable electronics, energy harvesters, bio-medical electronics, healthcare devices, advanced optical systems, and/or multimodal sensing systems.

## Methods

### Materials and fabrication of functional layers

All reagents were purchased from commercial sources and were used without further purification. The solution-processable PEDOT:PSS (Clevios P VP AI 4083, H.C. Starck Inc.), SY (PDY-132, Merck) semiconducting polymers and the silver nanowire (Ag-NW, NTC-01, Nanopyxis Co. Ltd.) suspension were used as received from the manufacturer.

For the vortex-spin-coating process, we used a truncated conical chuck made with Teflon. It had an inner top radius *R*_1_ of 17.25 mm, an inner bottom radius *R*_2_ of 7.25 mm, a height *H* of 10 mm, with a slant angle α of 45° with a maximum coating diameter of 14.5 mm (see Figs. 1 and S1). A substrate is attached to the bottom surface of the truncated conical chuck at its centre. A small volume of coating solution (~0.1–0.2 ml) of PEDOT:PSS or SY was introduced and used to fill the truncated conical chuck using a micropipette. After the coating solution had completely covered the substrate laid on the chuck, the chuck and substrate were rotated such that their rotation spreads the solution on the substrate by controlling the dimple shape of the vortex of the coating solution. The typical spinning speed ω for this step is in the range of 1000–6000 rpm, and it took approximately 1 min to form a film on the substrate.

To investigate the features of our solution-coating method, we tested two types of coating processes for the solution-processable functional layers: i) a functional layer fabricated by the conventional spin-coating method as a reference layer and ii) a functional layer fabricated by the vortex-spin-coating method as a sample layer. Surface morphology images of the fabricated functional layers were observed using non-contact atomic force microscopy (AFM, FlexAFM, Nanosurf AG). The microscopic morphologies and structures of the fabricated layers were also investigated using a non-contact 3D optical surface profiler (NV-2400, Nanosystem Co. Ltd.). The fabricated functional layers were also analysed by high-resolution scanning electron microscopy (SEM, Model JSM-6700F, JEOL Co.).

### Fabrication characterisation of the devices

A 3D OLED was fabricated using the functional layers coated by the vortex-spin-coating method on a bi-convex lens (Lens-OLEDs), as follows. A highly transparent silver nanowire (Ag-NW with an average diameter and length of about 25 nm and 32 μm, respectively, in isopropyl alcohol, 0.5–2.0 wt%) layer (thickness: ~60 nm, sheet resistance 30 ohm/square, transmittance ~ 90% at 550 nm), transferred by a well-established method onto a flat glass or bi-convex lens (diameter = 9.5 mm, focal length = 20 mm) substrate, was used as the transparent anode. After the transfer step, the Ag-NW anode on the substrate was treated with ultra-violet (UV) ozone for five minutes. A PEDOT:PSS layer was then vortex-spin-coated directly onto the Ag-NW anode to form a hole-injection layer (HIL) using an aqueous PEDOT:PSS solution. The PEDOT:PSS HIL was then annealed at 120 °C for 20 min. The thickness of the PEDOT:PSS layer was set to approximately 40 nm, as measured using a 3D surface profiler and non-contact AFM. For the light-emitting layer (EML), a solution of yellow-emitting SY was prepared by dissolving the SY polymer in toluene, which was then vortex-spin-coated onto the PEDOT:PSS HIL. The thickness of the SY EML was set to approximately 80 nm. Subsequently, an electron injection layer (EIL) of CsF (2 nm) and a cathode of Al (80 nm) were thermally deposited in a vacuum at a pressure of ~2.0 × 10^−4^ Pa using a vacuum chamber incorporated in a glovebox containing an inert atmosphere. Characterisation of the device was carried out at room temperature under ambient conditions, without encapsulation.

Current density-voltage-luminance (*J-V-L*) measurements of the fabricated OLEDs were taken at an ambient temperature using a computer-controlled chroma meter (CS-200, Minolta) with a source meter (Keithley 2400). The electroluminescent (EL) spectra of the devices were also obtained using a spectrometer (HR4000, Ocean Optics). The light-emitting area of the OLED on the lens substrate was estimated by a direct calculation with the image analysis software ImageJ (National Institutes of Health, USA).

## Supplementary information

supplementary figures
