# Supplementary material for: Uniform and bright light emission from a 3D organic light-emitting device fabricated on a bi-convex lens by a vortex-flow-assisted solution-coating method
Source: Sci Rep. 2019 Dec 3;9:18219. doi: 10.1038/s41598-019-54820-9 (PMC6890729; doi:10.1038/s41598-019-54820-9)
Supplement: Supplementary file 1 — supplementary figures [file 41598_2019_54820_MOESM1_ESM.pdf]

SUPPLEMENTARY INFORMATION

Uniform and bright light emission from a 3D organic light-emitting device fabricated on a bi-convex lens by a vortex-flow-assisted solution-coating method

Byoungchoo Park*, Seo Yeong Na, and In-Gon Bae

Department of Electrical and Biological Physics, Kwangwoon University, Wolgye-Dong, Nowon-gu, Seoul 139-701, Republic of Korea

*e-mail: bcpark@kw.ac.kr

(Supplementary Information)

1. Reference configuration of the system

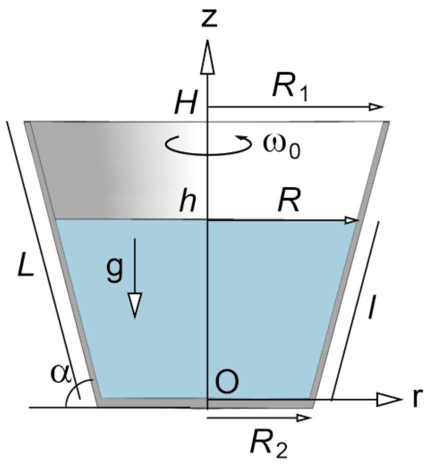

Supplementary Fig. S1. Reference configuration of a liquid in a rotating truncated cone with inner top radius R_1, inner bottom radius R_2, and height H with a slant angle of α and slant height of L with the relationship $H = L \sin \alpha$. The inner radius, height, and slant height of the liquid before its rise and without the capillary effects are R, h and l, respectively.

Supplementary Fig. S1 shows a reference configuration of the system, just at the point of the removal of the external torque which put the system into a rotating state. The reference configuration is virtual in the sense that it is assumed that the liquid has not yet begun to rise from its still level (h) state and that the capillary effects are frozen. The mass of the liquid is $m = \rho_l V_0$, where ρ_l is its density. The moment of inertia of the liquid in this state is $J_0 = \frac{3}{10} m \frac{(R^5 - {R_2}^5)}{(R^3 - {R_2}^3)}$, the moment of inertia of the rigid truncated conical container is $J_\bullet$, and $\omega_0 = \mathcal{H}(J_0 + J_\bullet)$ is the angular speed corresponding to the given angular momentum $\mathcal{H}$. The volume of the liquid is $V_0 = \frac{1}{3}\pi(R^2 + R \cdot R_2 + {R_2}^2) \cdot h$ and the surface area of the side wall of the truncated cone is $\pi(R + R_2) \cdot l$, where R, h, and l are the internal radius, height, and slant height ($l = \frac{h}{\sin \alpha} = h \csc \alpha$) of the liquid, respectively.

The internal energy U_0 associated with the reference liquid configuration, up to a constant term, is then expressed as

$$U_0 = \pi R^2 \sigma_{lv} + \pi(R + R_2) l \sigma_{sl} + \pi(R + R_1)(L - l)\sigma_{sv} + 2\pi R\tau + V_0 h_{CM} \gamma_l.$$

(S1.1)

Here, the solid/vapour, liquid/vapour, and solid/liquid surface energies are denoted by σ_{sv}, σ_{lv}, and σ_{sl}, respectively, and τ is the line tension along the triple solid/liquid/vapor contact line. The centre of mass of the liquid h_{CM} is $h_{CM} = \frac{R}{(R+R_2)} \cdot h$. The slant height of the truncated cone is L and $\gamma_l = \rho_l \cdot g$ is the specific weight of the liquid, where g is the acceleration of the gravity. The density of the surrounding gas is assumed to be much lower than the density of the liquid ($\rho_v \ll \rho_l$). It is assumed that the angular velocity ω_0 is sufficiently small such that the surface of the liquid does not reach the bottom of the truncated conical container, causing the interface energy $\pi {R_2}^2 \sigma_{sl}$ in the configuration shown in Supplementary Fig. S1 to be excluded from the expressions of Eq. (S1.1).

Next, the kinetic energy K_0 of the liquid in this configuration can be expressed as

$$K_0 = \frac{1}{2} J_0 {\omega_0}^2. \qquad \text{(S1.2)}$$

2. Axisymmetric shape of the liquid surface under uniform rotation: before the onset of dewetting at the bottom of the truncated cone

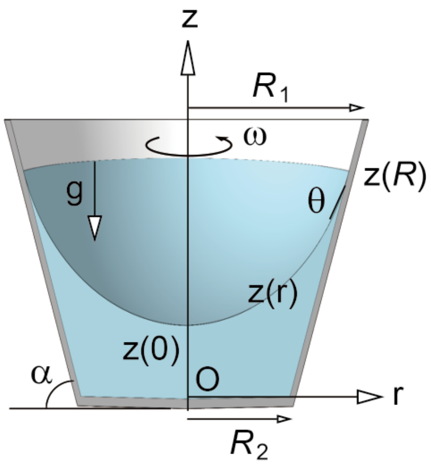

Supplementary Fig. S2. Axisymmetric shape of the liquid surface under uniform rotation with angular speed ω and angular momentum identical to that of the system in Supplementary Fig. S1.

After the removal of the external torque by which the truncated conical container was set in rotation, the liquid is in a state of rigid rotation around the z axis (Supplementary Fig. S2) with the angular velocity corresponding to the given angular momentum $\mathcal{H} = (J_0 + J_\bullet)\omega_0 = (J + J_\bullet)\omega$. Assuming that the shape of the liquid surface is axisymmetric and can be described by a single-valued function $z = z(\mathrm{r})$, the internal energy U of the system is

$$U = S\sigma_{lv} + \pi(R + R_2)z(R)\csc\alpha\,\sigma_{sl} + \pi(R + R_1)\big(H - z(R)\big)\csc\alpha\,\sigma_{sv} + 2\pi R\tau$$
$$+\gamma_l\pi\int_0^R r\cdot z^2\,dr - \gamma_l\pi\tan^2\alpha\int_{R_2}^R (r - R_2)^2 r\,dr. \tag{S2.1}$$

We neglect the small effect of gravity and the angular speed on the surface energy σ_{sv}. The corresponding kinetic energy K of the liquid is

$$K = \frac{1}{2}J\omega^2 = \pi\rho_l\omega^2\left[\int_0^R z\cdot r^3\,dr - \tan\alpha\int_{R_2}^R (r - R_2)r^3\,dr\right]. \tag{S2.2}$$

The area S in Eq. (S2.1) is the surface area of the liquid/vapour interface, whose profile is $z(r)$. The expressions of the surface area S and the volume V can be expressed as

$$S = 2\pi \int_0^R r(1+z'^2)^{1/2}\,dr,\ V = 2\pi \int_0^R z \cdot r\,dr - 2\pi \tan\alpha \int_{R_2}^R (r-R_2) r\,dr. \tag{S2.3}$$

Here, $(r,\ z)$ is the coordinate at an arbitrary point within the liquid. The effective mechanical potential of the system is $L = U - K$ and the appropriate functional for the variational study is $\Pi = L - \lambda V$, where λ is the Lagrangian multiplier (dimensionless pressure).

If a surface element dS at an arbitrary point of S, which is not on the bounding circle $r = R$, is assigned virtual displacement δu, then $\delta(dS) = -2\kappa \delta u_n dS$, where δu_n is the projection of δu onto the surface outward normal. The surface elements at the points of the contact circle $r = R$ must also be stretched in the direction tangential to the surface to preserve the liquid contact with its lateral wall. Hence, the total area change of the surface S can be written as

$$\delta S = -\int_S 2\kappa \delta u_n dS + 2\pi R \cos\theta\, \delta z(R). \tag{S2.4}$$

The variation of the potential function $\delta\Pi = \delta U - \delta K - \delta\lambda V$ thus becomes

$$\begin{aligned}\delta\Pi = &-\int_S \left[2\sigma_{lv}\kappa - \tfrac{1}{2}\gamma_l z(R) + \tfrac{1}{2}\rho_l \omega^2 r^2 + \lambda\right] \delta u_n dS \\ &+2\pi R \left(\sigma_{lv}\cos\theta + \tfrac{\csc\alpha}{2R}[\sigma_{sl}(R_2+R) - \sigma_{sv}(R_1+R)]\right)\delta z(R) \\ &+2\pi \begin{pmatrix} \tau - \tfrac{1}{2}\gamma_l \tan^2\alpha\ \ R(R-R_2)^2 + \tfrac{1}{2} z(R)\csc\alpha\,(\sigma_{sl} - \sigma_{sv}) \\ +\tfrac{1}{2}\rho_l\omega^2 \tan\alpha\ \ R^3(R-R_2) + \lambda \tan\alpha\ \ R(R-R_2) \end{pmatrix}\delta R.\end{aligned} \tag{S2.5}$$

In the gyrostatic configuration, the potential Π has a stationary value, and the expression in Eq. (S2.5) must vanish ($\delta\Pi = 0$). This leads to

$$2\pi\sigma_{lv}\cdot K(r) - \gamma_l\left[z(r) - \frac{\omega^2}{2g}r^2\right] + \lambda = 0, \tag{S2.6}$$

$$\sigma_{lv}\cos\theta + \frac{\csc\alpha}{2R}\left[\sigma_{sl}(R_2 + R) - \sigma_{sv}(R_1 + R)\right] = 0, \tag{S2.7}$$

$$\frac{z(R)}{\sin\alpha}(\sigma_{sv} - \sigma_{sl}) = 2\tau - \gamma_l\tan^2\alpha R(R - R_2)^2 + \rho_l\omega^2\tan\alpha R^3(R - R_2) + 2\lambda\tan\alpha R(R - R_2). \tag{S2.8}$$

Here, $K(r) = K(0) + \frac{\gamma_l}{2\sigma_{lv}}\left[z(r) - z(0) - \frac{\omega^2}{2g}r^2\right]$, describing the mean curvature of the liquid profile in the truncated conical container. The second equilibrium condition of Eq. (S2.7) yields the Young's equation for the contact angle,

$$\cos\theta = \frac{\csc\alpha}{2\sigma_{lv}}\left[\sigma_{sv}\left(\frac{R_1}{R} + 1\right) - \sigma_{sl}\left(\frac{R_2}{R} + 1\right)\right]. \tag{S2.9}$$

It should be noted the angle θ does not depend on g or ω because it is a local material property governed by the surface tensions alone, unaffected by the external fields due to gravity and rotation.

3. Axisymmetric shape of the liquid under uniform rotation: after the onset of dewetting

at the bottom of the truncated cone

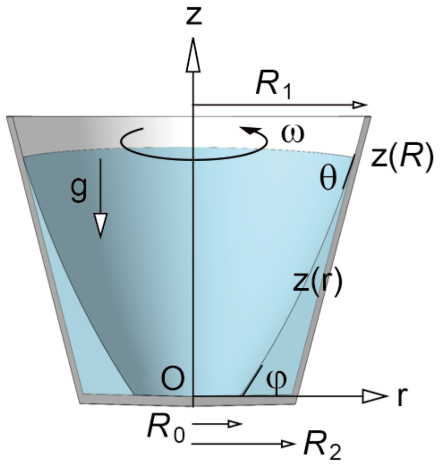

Supplementary Fig. S3. Liquid surface shape after the onset of dewetting, which has spread to the radius R_0. The wetting contact angles at the bottom and the lateral wall of the truncated conical container are φ and θ, respectively.

For a sufficiently high angular speed ω^*, the liquid surface will approach and touch the bottom of the truncated cone container; i.e., $z(0) = 0$. For an angular speed for which $\omega > \omega^*$, the liquid withdraws from the bottom surface of the container (Supplementary Fig. S3). On the microscopic scale, the withdrawal initially takes place due to the deposition of a thin liquid film at the bottom of the container. The film deposition process will continue unless the centrifugal force is able to rupture the molecular liquid bonds within the film, beyond which the actual dewetting of the bottom of the container could begin. Let $R_0 < R$ denote the extent of the deposited film or the actual dewetting. Again, the appropriate functional for the variational study is $\Pi = U - K - \lambda V$, with the Lagrangian multiplier λ. The free energy U is

$$U = S\sigma_{lv} + \pi z(R) \csc \alpha \left[\sigma_{sl}(R_2 + R) - \sigma_{sv}(R_1 + R)\right] + 2\pi R\tau + \pi {R_0}^2\sigma^* + 2\pi R_0\tau^*$$

$$+\frac{1}{2}\gamma_l\int_V z\,dV - \pi\gamma_l\tan^2\alpha\int_{R_2}^{R} r(r-R_2)^2 dr,$$

(S3.1)

and the kinetic energy is

$$K = \frac{1}{2}\rho_l\omega^2\int_V r^2\,dV - \pi\rho\omega^2\tan\alpha\int_{R_2}^{R} r^3(r-R_2)dr \tag{S3.2}$$

with

$$V = \int_V 1\,dV - 2\pi\tan\alpha\int_{R_2}^{R} r(r-R_2)dr. \tag{S3.3}$$

With regard to the actual dewetting, the surface energy σ^* is $\sigma^* = \hat{\sigma}_{sv} - \hat{\sigma}_{sl}$ with the surface energies $\hat{\sigma}_{sv}$ and $\hat{\sigma}_{sl}$ at bottom, which are different from the surface energies σ_{sv} and σ_{sl} at the lateral wall surface. The line tension along the triple contact line at the bottom surface is τ^*. If the liquid withdraws from the bottom surface of the container by leaving a thin film behind, the surface energy σ^* is equal to σ_{lv}. It is assumed that the film is sufficiently thick such that its lower face, which is in contact with the solid, can still be assigned surface energy σ_{sl}, while its upper face is assigned surface energy σ_{lv}. In this case, $\tau^* = 0$, as the liquid elevates from the film smoothly and without contact with a solid surface of the container.

The surface elements at the points of the contact circles $r = R_0$ and $r = R$ must also be stretched in the direction tangential to the surface to preserve the liquid contact with the bottom of the container and with its lateral wall. Thus, the total area change of the surface S can be written as

$$\delta S = -\int_S 2\kappa\delta u_n dS - 2\pi R_0\cos\varphi\,\delta R_0 + 2\pi R\cos\theta\,\delta z(R). \tag{S3.4}$$

The variation of the potential function $\delta\Pi$ becomes

$$\delta\Pi = -\int_S \left(\sigma_{lv}2\kappa - \frac{1}{2}\gamma_l z + \frac{1}{2}\rho\omega^2 r^2 + \lambda\right)\delta u_n dS + 2\pi R_0 \left(\sigma^* + \frac{\tau^*}{R_0} - \sigma_{lv}\cos\varphi\right)\delta R_0$$

$$+2\pi R\left(\sigma_{lv}\cos\theta + \frac{\csc\alpha}{2R}[\sigma_{sl}(R_2+R) - \sigma_{sv}(R_1+R)]\right)\delta z(R)$$

$$+2\pi\begin{pmatrix}\tau - \frac{1}{2}\gamma_l\tan^2\alpha \ \ R(R-R_2)^2 + \frac{1}{2}z(R)\csc\alpha\,(\sigma_{sl}-\sigma_{sv}) \\ +\frac{1}{2}\rho_l\omega^2\tan\alpha \ \ R^3(R-R_2) + \lambda\tan\alpha \ \ R(R-R_2)\end{pmatrix}\delta R.$$

(S3.5)

The stationary condition $\delta\Pi = 0$ leads to

$$2\sigma_{lv}\kappa - \frac{1}{2}\gamma_l z + \frac{1}{2}\rho_l\omega^2 r^2 + \lambda = 0, \quad \text{(S3.6)}$$

$$\sigma^* - \sigma_{lv}\cos\varphi + \frac{\tau^*}{R_0} = 0, \quad \text{(S3.7)}$$

$$\sigma_{lv}\cos\theta + \frac{\csc\alpha}{2R}[\sigma_{sl}(R_2+R) - \sigma_{sv}(R_1+R)] = 0, \quad \text{(S3.8)}$$

$$\frac{z(R)}{\sin\alpha}(\sigma_{sv} - \sigma_{sl}) =$$

$$2\tau - \gamma_l\tan^2\alpha R(R-R_2)^2 + \rho_l\omega^2\tan\alpha R^3(R-R_2) + 2\lambda\tan\alpha R(R-R_2).$$

(S3.9)

The two expressions of Eqs. (S3.7) and (S3.8) specify the wetting angles φ and φ as

$$\cos\varphi = \frac{\sigma^*}{\sigma_{lv}} + \frac{\tau^*}{\sigma_{lv}R_0}, \quad \text{(S3.10)}$$

$$\cos\theta = \frac{\csc\alpha}{2\sigma_{lv}}\left[\sigma_{sv}\left(\frac{R_1}{R}+1\right) - \sigma_{sl}\left(\frac{R_2}{R}+1\right)\right]. \quad \text{(S3.11)}$$

Eq. (S3.10) defines the contact angle φ between the liquid and the bottom of the truncated cone and Eq. (S3.11) is Young's equation for the contact angle θ between the liquid and the

lateral wall. If a thin film is deposited behind a withdrawing liquid, then

$$\sigma^* = \sigma_{lv} \text{ and } \tau^* = 0, \tag{S3.12}$$

such that Eq. (S3.10) results in

$$\varphi = 0; \tag{S3.13}$$

that is, the liquid elevates from a deposited film with a vanishing slope, i.e.,

$$z'(R_0) = 0. \tag{S3.14}$$

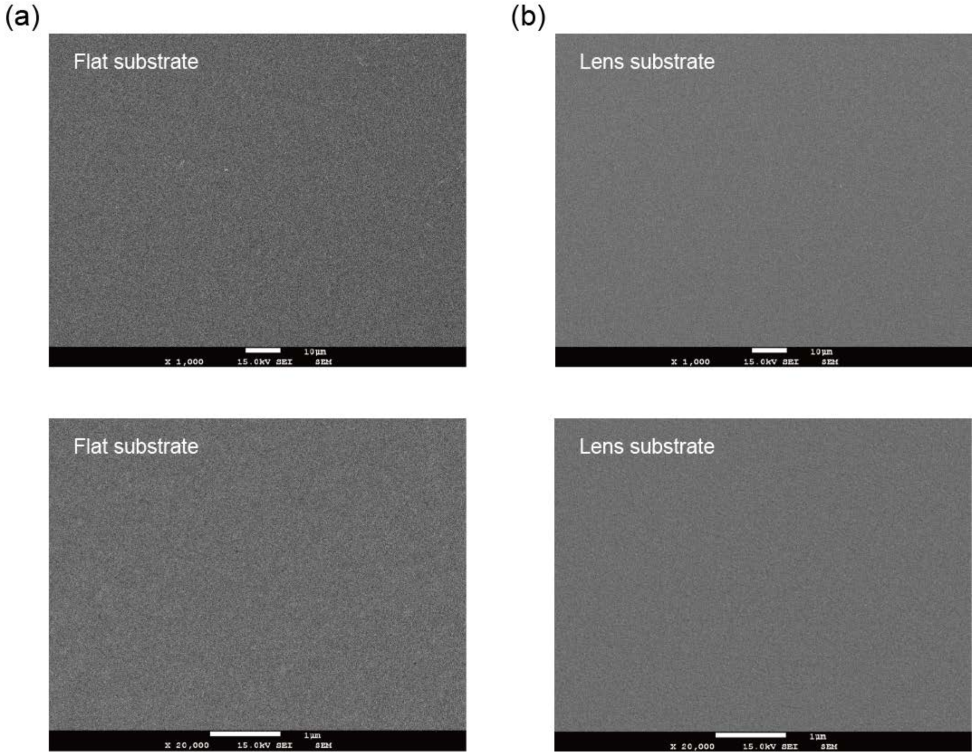

Supplementary Fig. S4. SEM images of vortex-spin-coated SY polymer layers on bi-convex lens substrates. (**a**) SEM images of a typical spin-coated SY polymer layer on a flat substrate, and (**b**) SEM images of a vortex-spin-coated SY polymer layer on a bi-convex lens. The scale bars in the upper and lower panels are 10 μm and 1 μm, respectively.

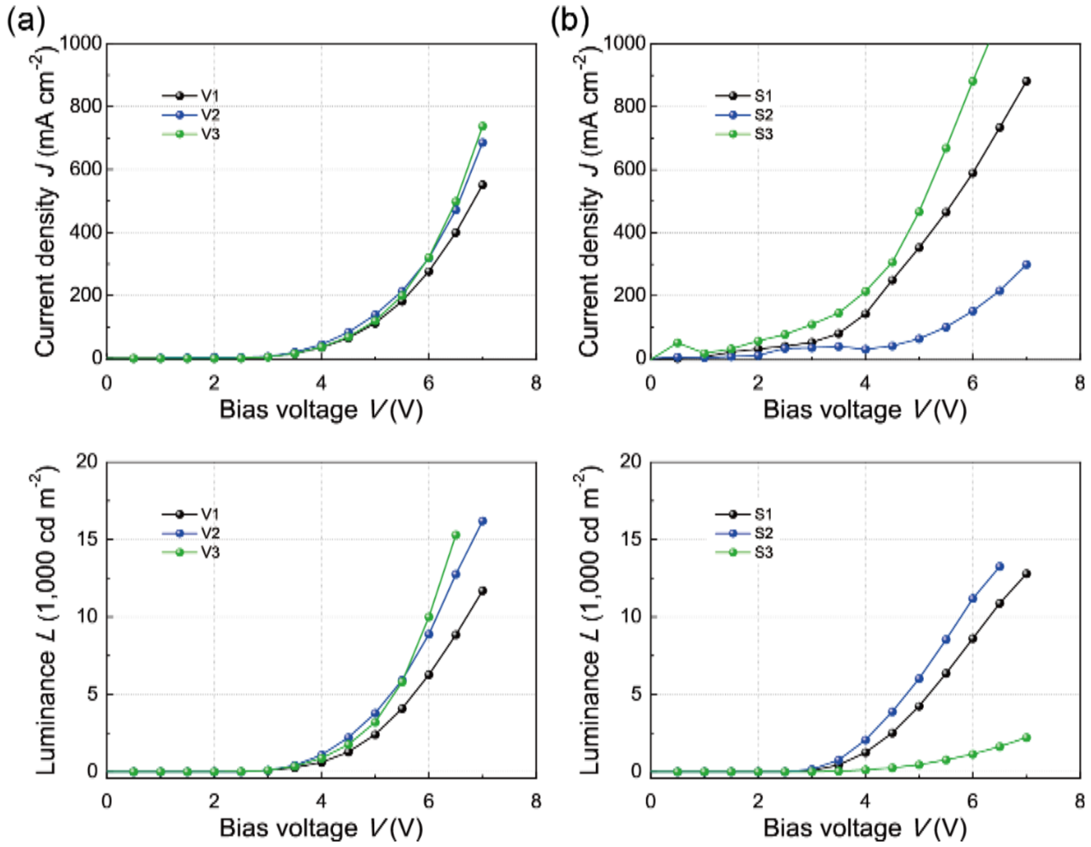

Supplementary Fig. S5. Variations of the current density-voltage (*J*-*V*) and luminance-voltage (*L*-*V*) characteristics of OLEDs fabricated by the vortex-spin-coating method and the conventional spin-coating method on bi-convex lens substrates. *J*-*V* (upper) and *L*-*V* (lower) characteristics of the three OLEDs fabricated by the vortex-flow-assisted spin-coating method (**a**) and by the conventional spin-coating method (**b**) on the bi-convex lens substrates.
